# The Effect of the COVID-19 Pandemic on Patient Selection, Surgical Procedures, and Postoperative Complications in a Specialized Dental Implant Clinic

**DOI:** 10.3390/jcm11030855

**Published:** 2022-02-06

**Authors:** Balazs Feher, Cordelia Wieser, Theresa Lukes, Christian Ulm, Reinhard Gruber, Ulrike Kuchler

**Affiliations:** 1 Department of Oral Surgery, University Clinic of Dentistry, Medical University of Vienna, 1090 Vienna, Austria; christian.ulm@meduniwien.ac.at (C.U.); ulrike.kuchler@meduniwien.ac.at (U.K.); 2 Department of Oral Biology, University Clinic of Dentistry, Medical University of Vienna, 1090 Vienna, Austria; reinhard.gruber@meduniwien.ac.at; 3 Department of Dental Training, University Clinic of Dentistry, Medical University of Vienna, 1090 Vienna, Austria; n1445032@students.meduniwien.ac.at (C.W.); n1242565@students.meduniwien.ac.at (T.L.); 4 Austrian Cluster for Tissue Regeneration, Ludwig Boltzmann Institute for Experimental and Clinical Traumatology, 1200 Vienna, Austria; 5 Department of Periodontology, School of Dental Medicine, University of Bern, 3012 Bern, Switzerland

**Keywords:** dentistry, surgery, oral, dental implantation, retrospective studies, population characteristics

## Abstract

During the coronavirus disease 2019 (COVID-19) pandemic, aerosol-generating procedures, including dental implant treatments, are considered high-risk. With dental implant treatment mostly an elective procedure, we aimed to assess whether the pandemic influenced patient selection, surgical procedures, and postoperative complications. We compared dental implant treatments during (March to December 2020) and before (December 2018 to February 2020) the COVID-19 pandemic based on patient and implant parameters, as well as postoperative complications. For analysis, we used the Chi-squared test with the Holm–Sidak correction for multiple comparisons. The number of implants placed during the COVID-19 pandemic (696 implants in 406 patients, 70 implants per month) was comparable to pre-pandemic levels (1204 implants in 616 patients, 80 implants per month). Regarding patient parameters, there were no significant differences in respiratory (*p* = 0.69) and cardiovascular conditions (*p* = 0.06), diabetes (*p* = 0.69), and smoking (*p* = 0.68). Regarding implant parameters, there was a significant difference in the distribution of augmentative procedures (no augmentation, guided bone regeneration, and sinus floor elevation, *p* = 0.01), but no significant differences in the types of edentulous spaces (*p* = 0.19) and the timing of implant placement (*p* = 0.52). Regarding complications, there were significantly fewer minor complications (*p* < 0.001) and early (i.e., before loading) implant failures (*p* = 0.02) compared with pre-pandemic levels. Our results suggest that the COVID-19 pandemic had no effect on patient selection and only a slight effect on the surgical procedures. However, postoperative complications, including early failures, were significantly less prevalent during the pandemic.

## 1. Introduction

The airborne transmission of severe acute respiratory syndrome coronavirus 2 (SARS-CoV-2), the RNA virus that causes coronavirus disease 2019 (COVID-19), poses a considerable challenge in the dental setting, where patients are unable to wear masks or other facial barriers while in close contact to the personnel providing care [1]. Furthermore, aerosol-generating procedures, including dental implant placement, are generally considered high-risk as they can produce and spread contaminated droplets [2,3,4,5]. Surface contamination through these droplets is substantial at close proximity and still detectable at a maximum distance of 4 m from the source [6]. Consequently, multiple guidelines have recommended prioritizing no-aerosol over aerosol-generating procedures (e.g., using manual over rotary or ultrasonic instruments [1]) or postponing aerosol-generating procedures of potentially infectious patients or patients at an increased risk for COVID-19 entirely, especially if these procedures are elective [7,8,9,10].

In general, dental implant treatment is an elective procedure. Nevertheless, implant placement cannot be postponed indefinitely, as edentulous bone is subject to catabolic dimensional changes over time [11]. In addition, there is a highly important quality-of-life aspect to dental implant treatment [12,13]. Thus, the clinical decision-making process during the COVID-19 pandemic must balance this increase in quality of life against a potentially increased risk of SARS-CoV-2 transmission. This could affect both patient selection and surgical procedures. Patients with relevant comorbidities (e.g., respiratory and cardiovascular conditions [14], diabetes [15]) might choose to—or be advised to—postpone their dental implant treatment until such time that they are at no increased risk for SARS-CoV-2 transmission. In addition, oral surgeons might choose to limit the number and complexity of procedures (e.g., restricting augmentative procedures to where indisputably necessary) to minimize the patients’ and their own risk of transmission.

Previous work has focused on various guidelines, preventive measures, as well as the epidemiological aspect of dental treatments during the COVID-19 pandemic [8]. However, the effect of the pandemic on the treatments themselves remains elusive. The aim of this study was therefore to retrospectively assess whether the COVID-19 pandemic had an effect on patient selection, surgical procedures, and postoperative complications.

## 2. Materials and Methods

This is a retrospective study designed and conducted in accordance with the Declaration of Helsinki and its subsequent revisions [16]. The study protocol was approved by the institutional review board of the Medical University of Vienna (No. 1282/2021). We reviewed and extracted data from the electronic patient records (Medfolio, Nexus, Donaueschingen, Germany) of our clinic. We included patients that received at least one dental implant at our clinic between 1 December 2018 and 1 December 2020. Data were extracted into a standardized patient–feature matrix by two researchers (CW, TL) and subsequently error-proofed by two different researchers (BF, UK) in an independent manner. Only complete data were used in this study. Results are reported in accordance with STROBE criteria for observational studies [17]; the complete checklist is available as a Supplementary File.

Patient parameters included demographic data and medical history. They comprised age, sex, smoking status, comorbidities (e.g., respiratory conditions, cardiovascular conditions, diabetes mellitus), implant location, and type of edentulous space (single-tooth gap, extended gap, distal extension, edentulous jaw). We considered the following implant parameters: length, diameter, bone augmentation (guided bone regeneration or sinus floor elevation), and timing of implant placement (immediate, early, or late [18]). In addition, we assessed minor postoperative complications (e.g., bleeding, suppuration, swelling, local infection, hematoma, temporary neurosensory disturbance), as well as early implant failure (i.e., before loading [19]).

The sample for the statistical analysis included every patient who received a dental implant between 1 December 2018 and 1 December 2020, for which complete data were available in the electronic patient records. We first collected all data and checked them for possible errors. The dataset was then split into a sample before the pandemic (control sample, December 2018 to February 2020) and a sample during the pandemic (test sample, March 2020 to December 2020). We analyzed all parameters in a descriptive manner. We further compared the prevalence and distributions of smoking, respiratory conditions, cardiovascular conditions, types of gaps, bone augmentation, timing of implant placement, minor postoperative complications, as well as early implant failures using the Chi-squared test. To correct for multiple comparisons, we used the Holm–Bonferroni correction with the Sidak modification. We set the level of significance at α < 0.05. Statistical analysis was performed by one researcher (BF) using Prism 9.2.0 (GraphPad Software, La Jolla, CA, USA).

## 3. Results

### 3.1. Sample Characteristics

The sample during the pandemic included 406 patients (median age at first implantation: 54.0 years, interquartile range: 41.9–62.2, 56% female, 16% smokers) and 696 implants (70 implants per month, 54% maxilla). The number of implants per patient ranged from 1 (*n* = 236) to 10 (*n* = 1), with an average of 1.7 implants per patient. The mean implant diameter was 4.1 ± 0.5 mm (standard deviation). The mean implant length was 11.0 ± 1.3 mm.

**Figure 1:**
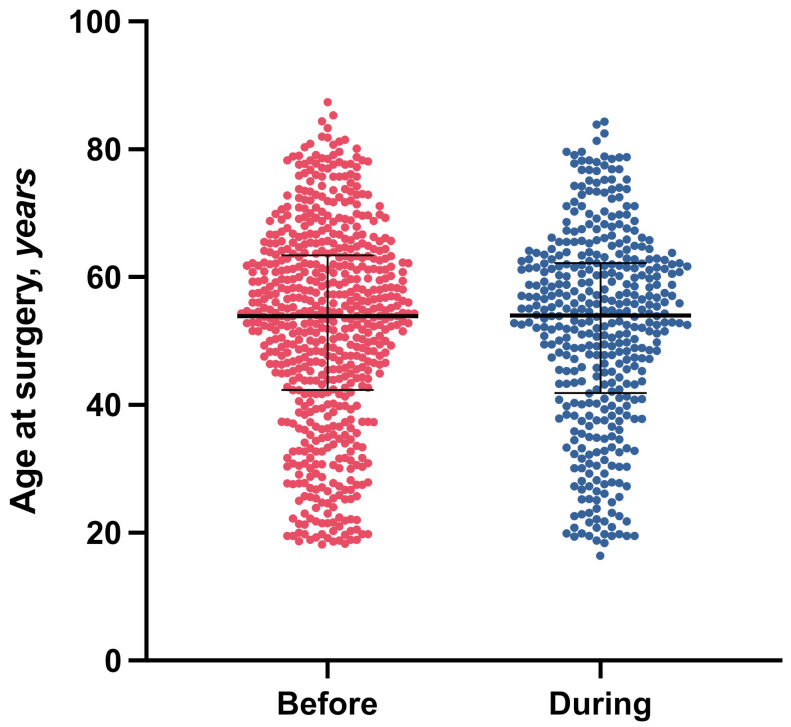
Age distribution. Bars represent medians and interquartile ranges.

The sample before the pandemic included 616 patients (median age at first implantation: 54.0 years, interquartile range: 42.5–63.5, 53% female, 19% smokers) and 1204 implants (80 implants per month, 47% maxilla). The number of implants per patient ranged from 1 (*n* = 320) to 11 (*n* = 2), with an average of 2.0 implants per patient. The mean implant diameter was 4.1 ± 0.5 mm. The mean implant length was 11.0 ± 1.2 mm. A detailed description of the samples is shown in Table 1, and age distributions are shown in Figure 1.

### 3.2. Patient Parameters

**Figure 2:**
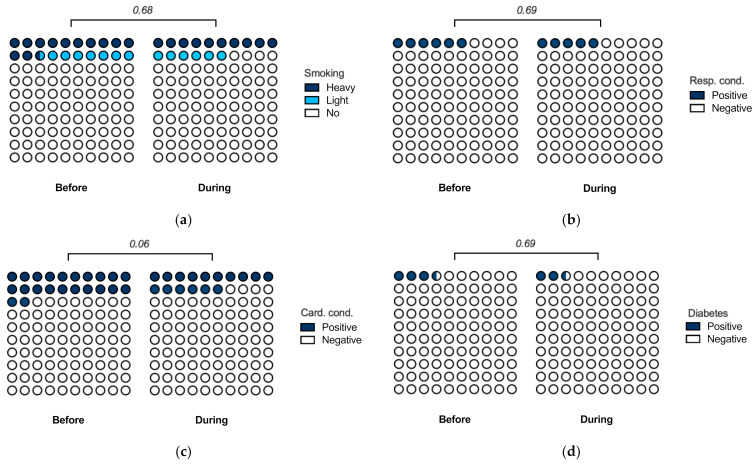
Patient parameters. (**a**) Smoking. Patients were considered light smokers if they smoked fewer than 10 cigarettes per day. (**b**) Respiratory conditions. Positive if patient history included asthma (International Classification of Diseases [ICD] 11 code CA23), chronic obstructive pulmonary disease (CA22), or pulmonary embolism (BB00). (**c**) Cardiovascular conditions. Positive if patient history included hypertension (BA00), hypotension (BA2Z), arrythmia (BC80, BC81), thrombosis/thromboembolism (BD71, BD72), or cardiac/vascular transplants/grafts (QB50). (**d**) Diabetes (5A14). All *p*-values using the Chi-squared test with the Holm–Sidak correction for multiple testing.

First, we compared patient parameters between the samples. There were 339 non-smokers (84%), 24 light smokers (fewer than 10 cigarettes per day, 6%), and 41 heavy smokers (at least 10 cigarettes per day, 10%) during the pandemic, compared to 493 non-smokers (80%), 46 light smokers (7%), and 76 heavy smokers (12%) before the pandemic (*p* = 0.68) (Figure 2a). Respiratory conditions were reported by 21 patients (5%) during the pandemic, compared to 37 patients (6%) before the pandemic (*p* = 0.69) (Figure 2b). Cardiovascular conditions were reported by 64 patients (16%) during the pandemic, compared to 137 patients (22%) before the pandemic (*p* = 0.06) (Figure 2c). Diabetes was reported by 11 patients (3%) during the pandemic, compared to 22 patients (4%) before the pandemic (*p* = 0.69) (Figure 2d). Taken together, the data suggest that the populations treated during and before the pandemic were largely comparable. While there were fewer cardiovascular conditions reported during the pandemic, this difference was not significant.

### 3.3. Implant Parameters

**Figure 3:**
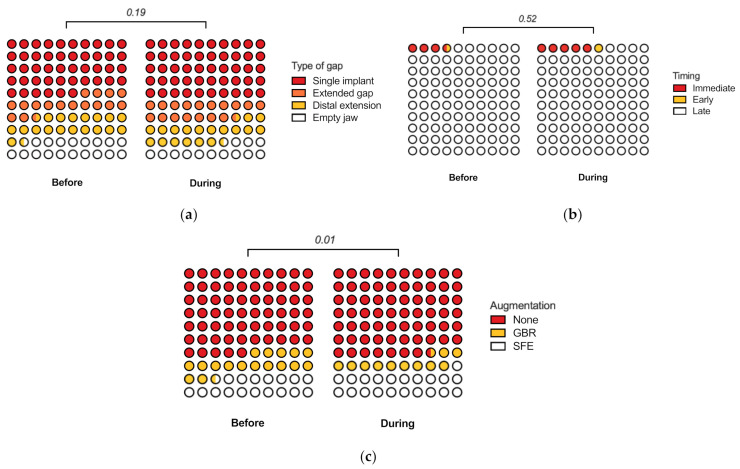
Implant parameters. (**a**) Type of edentulous space. (**b**) Timing of implant placement. Immediate implant placement took place in the same surgery as tooth extraction. Early implant placement took place no later than 8 weeks following tooth extraction. (**c**) Bone augmentation. *GBR*—guided bone regeneration; *SFE*—sinus floor elevation. All *p*-values using the Chi-squared test with the Holm-Sidak correction for multiple testing.

Next, we compared implant parameters between the samples. There were 348 single implants (50%), 121 implants placed in extended edentulous gaps (17%), 132 implants placed as distal extensions (19%), and 95 implants placed in empty jaws (14%) during the pandemic, compared to 556 single implants (46%), 198 implants placed in extended edentulous gaps (16%), 225 implants placed as distal extensions (19%), and 225 implants placed in empty jaws (19%) before the pandemic (*p* = 0.19) (Figure 3a). There were 34 immediate implants (5%), 9 implants placed early (1%), and 653 implants placed late (94%) during the pandemic, compared to 41 immediate implants (3%), 10 implants placed early (1%), and 1153 implants placed late (96%) before the pandemic (*p* = 0.52) (Figure 3b).

There were 469 implants placed without bone augmentation (67%), 82 implants placed with guided bone regeneration (12%), and 145 implants placed with sinus floor elevation (21%) during the pandemic, compared with 783 implants placed without bone augmentation (35%), 213 implants placed with guided bone regeneration (18%), and 208 implants placed with sinus floor elevation (17%) before the pandemic (*p* = 0.01) (Figure 3c). Taken together, the data suggest a significant difference in the use of bone augmentation, but no differences were found between the types of edentulous spaces as well as the timing of implant placements performed during and before the pandemic.

### 3.4. Postoperative Complications

**Figure 4:**
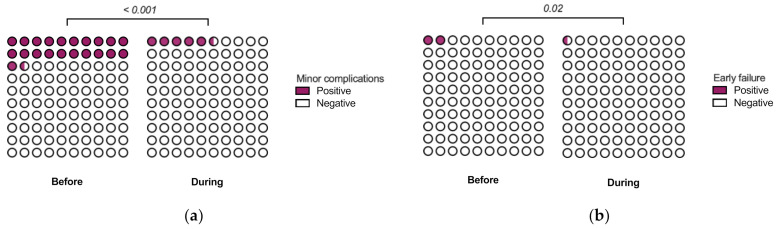
Postoperative complications. (**a**) Minor complications. Positive if patient bleeding, suppuration, swelling, local infection, hematoma, or temporary neurosensory disturbance was either reported by the patient or observed during a follow-up visit. (**b**) Early (i.e., before loading) implant failures. All *p*-values using the Chi-squared test with the Holm–Sidak correction for multiple testing.

Finally, we compared postoperative complications between the sample. Minor complications included bleeding or suppuration, swelling, local infection, hematoma, as well as temporary neurosensory disturbance. These occurred in 23 patients (6%) and affected 42 (6%) of the implants during the pandemic, compared to 133 patients (22%) and 261 implants (22%) before the pandemic (*p* < 0.001 at the patient level) (Figure 4a). A total of 3 implants failed before loading (<1%) during the pandemic, compared to 26 implants (2%) before the pandemic (*p* = 0.02) (Figure 4b). Taken together, the data suggest a significantly lower prevalence of postoperative complications, including early implant failure, between treatments during and before the pandemic.

## 4. Discussion

We studied dental implant treatments during the COVID-19 pandemic and compared them to a pre-pandemic control population with regards to patient and implant factors as well as postoperative complications. First, we found that the patient populations treated during and before the pandemic were largely comparable. While there were fewer cardiovascular conditions reported during the pandemic, this difference was not significant. Second, our data suggested a significant difference in the use of bone augmentation but no differences between the types of edentulous spaces as well as timing of implant placements performed during and before the pandemic. Third, we observed significantly fewer postoperative complications, including early implant failure, between treatments during and before the pandemic. Overall, the data show that the COVID-19 pandemic had virtually no effect on most aspects of dental implant treatment. Nonetheless, both minor postoperative complications and early implant failures were significantly less prevalent during the pandemic.

Our findings with regards to patient selection and surgical procedures relate well to others who found that after mostly restricting their work to emergency care in the initial phase of the pandemic, dental health care professionals resumed elective care [5]. Notably, the consideration to return to elective surgery soon after the first wave of the pandemic can also be observed in other surgical fields (e.g., cardiac [20], orthopedic [21,22], and plastic [23] surgery). Notably, ours is among the first studies to provide a retrospective analysis reporting on a large patient cohort treated during the pandemic. Importantly, with very strict measures in place to prevent transmission of SARS-CoV-2, there have been to date no clusters among patients or providers. This is also in line with recent work from other surgical fields showing no increased number of infections following elective surgery [24,25].

The clinical relevance of our findings is threefold. First, the data suggest that with adequate preventive measures in place, the benefits of dental implant treatment for patients outweighed their risk of SARS-CoV-2 transmission. It appears that postponing their dental implant treatment had too high an opportunity cost for patients. This is an important finding with regards to the quality-of-life increase patients expect from a dental implant treatment. Second, the data suggest much fewer postoperative complications and early implant failures. We believe the findings with regards to postoperative complications should be interpreted with caution. While all pre-pandemic dental implants had already been placed under sterile conditions, additional personal protective equipment (e.g., filtering face pieces) might have played a beneficial role in preventing surgical site infections. It is further possible that patients wanted to minimize the number of visits to our clinic during the pandemic and therefore chose to not report minor complications. While patients might have decided to undergo dental implant surgery notwithstanding the risk of SARS-CoV-2 transmission, they might not have made the same decision for a postoperative check-up visit. In comparison, early implant failure is not a subjective complication. Notably, 3 out of 696 implants failed before loading during the pandemic, compared with 26 out of 1204 implants before the pandemic, making the early failure rate 75% lower during the pandemic. While it is difficult to explain this finding, we believe its clinical relevance is unquestionable. One possible explanation is the overall lower prevalence of comorbidities and extensive augmentative procedures, albeit these differences were not significant in this study. Nonetheless, it should be noted that in our previous work using a dataset of over 2400 implants in over 1100 patients, neither comorbidities nor surgical procedures could accurately predict early implant failure [26]. Third, the data suggest that datasets containing dental implant treatments during the pandemic are comparable to pre-pandemic datasets, allowing their use for training or validation of statistical models. Prior to our study, this would not have been possible as we could not know whether the patient population can be considered homogenous.

The main limitation of our study is its potentially limited generalizability due to its sample being from a single institute in one location, focusing on a subset of oral surgical procedures using endosseous implants. It remains to be assessed whether other specialized implant clinics observed similarly unchanged tendencies with regards to patient selection and surgical procedures, especially clinics offering different implant types (e.g., zygomatic or subperiostal implants). It further remains to be assessed whether our findings are relatable to dental procedures, especially elective treatments, where the time component is not as relevant as in dental implant treatment (i.e., cosmetic dentistry). Further research should consider collating large datasets from different sources and including other dental treatments. However, the inclusion of new features must be balanced against maintaining a high quality of the patient–feature matrix to not compromise the analysis.

## 5. Conclusions

Within the limitations of this study, the results suggest that the COVID-19 pandemic had no effect on patient selection and only a slight effect on the surgical procedures. Nonetheless, postoperative complications, including early implant failure, were significantly less prevalent during the pandemic.

## Figures and Tables

**Table 1:** Subject and implant characteristics.

	Before the Pandemic		During the Pandemic	
Patient Parameters	Patients, *n* (%)	Implants, *n* (%)	Patients, *n* (%)	Implants, *n* (%)
Sex				
Female	324 (53)	646 (54)	227 (56)	382 (55)
Male	292 (47)	558 (46)	179 (44)	314 (45)
Smoking				
Non-smoker	493 (80)	941 (78)	339 (84)	583 (84)
Light smoker (<10 d^−1^)	46 (7)	88 (7)	24 (6)	32 (5)
Heavy smoker (≥10 d^−1^)	76 (12)	175 (15)	41 (10)	81 (12)
Comorbidities				
Respiratory conditions ^1^	37 (6)	68 (6)	21 (5)	51 (7)
Cardiovasc. Conditions ^2^	137 (22)	312 (26)	64 (16)	106 (15)
Diabetes	22 (4)	50 (4)	11 (3)	25 (4)
**Implant Parameters**		**Implants, *n* (%)**		**Implants, *n* (%)**
Treatment indication				
Single implant		556 (46)		348 (50)
Extended gap		198 (16)		121 (17)
Distal extension		225 (19)		132 (19)
Empty jaw		225 (19)		95 (14)
Timing				
Immediate		41 (3)		34 (5)
Early		10 (1)		9 (1)
Late		1153 (96)		653 (94)
Augmentation technique				
GBR ^3^		213 (18)		82 (12)
Sinus floor elevation		208 (17)		145 (21)
None		783 (35)		469 (67)

^1^ Positive if patient history included asthma (International Classification of Diseases [ICD] 11 code CA23), chronic obstructive pulmonary disease (CA22), or pulmonary embolism (BB00). ^2^ Positive if patient history included hypertension (BA00), hypotension (BA2Z), arrythmia (BC80, BC81), thrombosis/thromboembolism (BD71, BD72), or cardiac/vascular transplants/grafts (QB50). ^3^ Guided bone regeneration.

## Data Availability

Data supporting the reported findings are available from the corresponding author upon reasonable request.

## Supplementary Materials

The following supporting information can be downloaded at: https://www.mdpi.com/article/10.3390/jcm11030855/s1.
